# Typhlocolitis associated with *Clostridium difficile* ribotypes 078 and 110 in neonatal piglets from a commercial Irish pig herd

**DOI:** 10.1186/s13620-016-0070-9

**Published:** 2016-08-19

**Authors:** Máire C. McElroy, Martin Hill, Geraldine Moloney, Micheál Mac Aogáin, Shane McGettrick, Áine O’Doherty, Thomas R. Rogers

**Affiliations:** 1 Pathology Division, Central Veterinary Research Laboratory, Backweston Campus, Celbridge, Co. Kildare Ireland; 2 Department of Clinical Microbiology, Trinity College Dublin, Dublin, Ireland

**Keywords:** *Clostridium difficile*, Typhlocolitis, Pigs, PCR ribotyping

## Abstract

**Background:**

*Clostridium difficile* is a recognised cause of typhlocolitis and diarrhoea in neonatal pigs but has never been confirmed in association with pathology and disease in Irish pigs.

**Case Presentation:**

Four neonatal piglets, with a history of diarrhoea were referred to the Central Veterinary Research Laboratory, Backweston for necropsy. They were from a fully integrated, commercial pig farm with approximately 1000 sows. Three piglets had acute, superficial, erosive and suppurative typhlocolitis and the other had mild suppurative mesocolitis. *Clostridium difficile* (*C. difficile*) toxins A/B were detected using ELISA in the colonic contents from each piglet. *C. difficile* isolates from two of the piglets were PCR-ribotyped as 078 and an isolate from a third pig was ribotyped as 110.

**Conclusions:**

This is the first report confirming *C. difficile* in association with typhlocolitis in Irish pigs.

## Background

*Clostridium difficile* is a toxin producing, Gram-positive, spore-forming, anaerobic enteropathogen of humans and animals. It has recently emerged as a major cause of porcine neonatal diarrhoea in America [1]. It has also been reported in association with neonatal diarrhoea in Europe although one study found no clear association between *C. difficile* isolation and diarrhoea [2, 3]. Porcine *Clostridium difficile* -associated disease (CDAD) typically manifests itself as early-onset diarrhoea and sudden death in piglets 1–7 days of age. Gross lesions may include mesocolonic oedema and large intestines may be filled with pasty to watery yellowish contents. Histopathological mucosal lesions are limited to the caecum and colon. They are typically mild, but vary from grossly inapparent, multifocal necrosis of surface epithelial cells to transmural necrosis. The classic lesions are segmental erosions of the epithelium with effusion of fibrin and neutrophils into the lumen, so-called “volcano ulcers” [4].

CDAD occurs when *C. difficile* proliferates after endogenous intestinal flora is disrupted, either by a change in diet or antimicrobial treatment [5]. *C. difficile* produces two major toxins, Toxin A (TcdA) and Toxin B (TcdB) that act synergistically to cause apoptosis of mucosal epithelial cells and disruption of intracellular actin filaments responsible for cell to cell adhesion. Consequently, there is increased permeability of mucosal surfaces. Toxins A and B also initiate an inflammatory cascade that can result in increased damage to host tissues and fluid exudation [6]. The requirements for development of CDAD are disruption of normal intestinal or colonic flora, presence of the organism in the environment, and the production of toxins [5].

The standard for diagnosis of porcine CDAD is detection of toxins A and B in faeces or colonic contents, generally using commercially available enzyme immunoassays. Cultivation of *C. difficile* is difficult to interpret because it can be found in healthy pigs, therefore its isolation may have little diagnostic relevance [4].

*C. difficile* is also one of the most important nosocomial pathogens of humans, primarily associated with intestinal dysbiosis due to antibiotic administration. In recent years the epidemiology of human disease is changing with more community-acquired infections and emergence of strains in humans that are common in domestic animals [5]. Therefore, considerable interest is developing in potential zoonotic capabilities of *C. difficile.*

The aims of this paper are to document typhlocolitis associated with *C. difficile* in an outbreak of diarrhoea in neonatal pigs from a commercial pig farm in Ireland and to report the strain typing results of the *C. difficile* isolates.

## Case presentation

Four piglets, 3-4-days old, that had died during an outbreak of high morbidity, low mortality, neonatal diarrhoea in a 1000 sow commercial pig herd were submitted for necropsy. Details of treatment prior to death were not available.

At gross necropsy all four carcasses were well preserved with adequate body fat reserves. Stomachs were filled with milk. There was mild mesocolonic oedema, and small intestinal and colonic contents were soft and yellow in all four. No gross changes were noted in intestinal, caecal or colonic mucosa. Other body systems were unremarkable.

### Histopathology

At least 6 sections from representative areas along the length of the small intestine, a section of caecum and at least six sections of spiral colon were sampled for histopathology. They were fixed in buffered formalin, processed routinely and stained with haematoxylin and eosin.

**Fig. 1:**
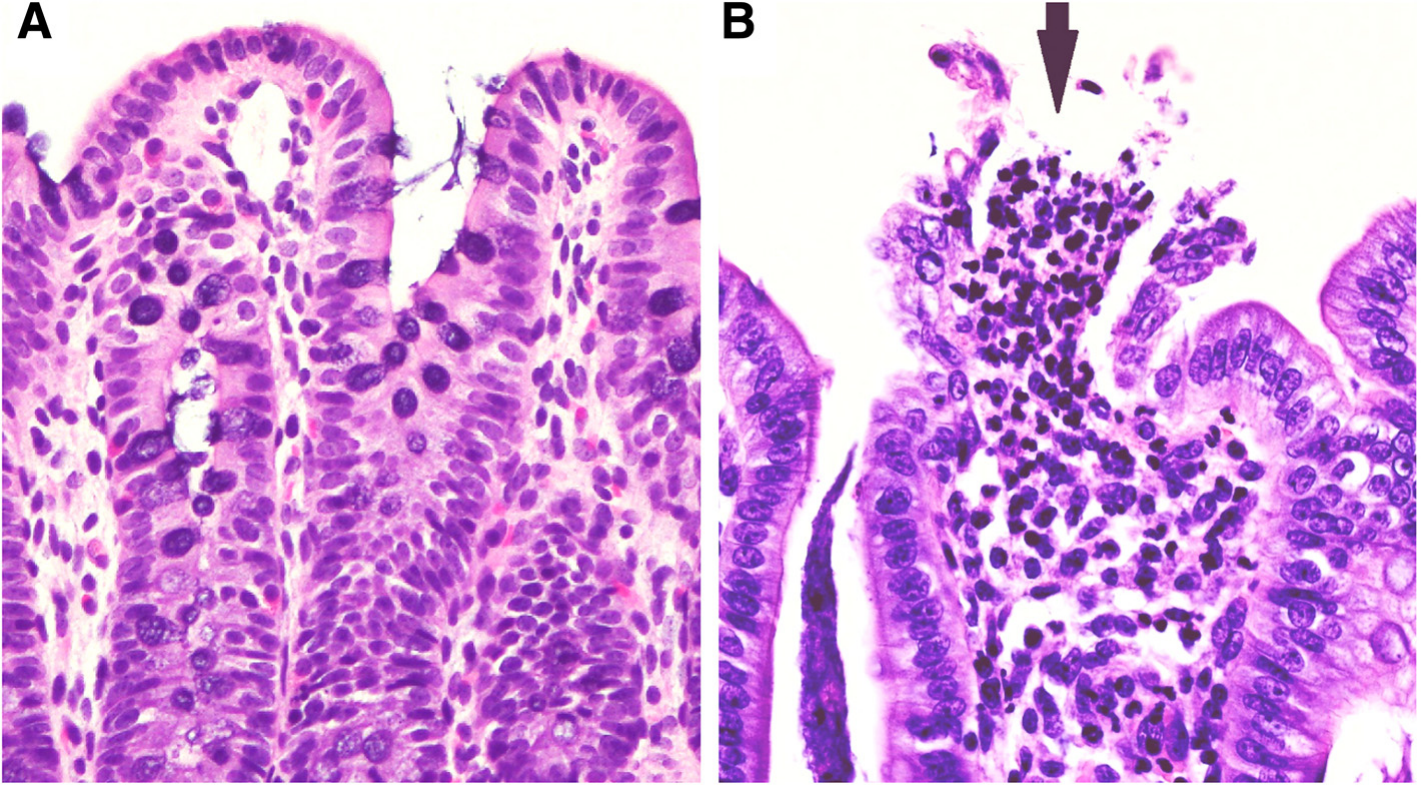
Pig. Colon. **a** Normal colon lined by columnar epithelial cells. **b** Superficial, erosive colitis with infiltration of neutrophils into the lamina propria and effusion into the lumen – ‘volcano lesion’ (*arrow*) (Haematoxylin and eosin 20×)

On histopathological examination three of the piglets had mild (*n* = 1) to severe (*n* = 2), multifocal, superficial fibrinosuppurative and erosive colitis with neutrophils and fibrin spilling from lamina propria through the eroded epithelium and into the lumen (‘volcano lesions’) (Fig. 1). In the other piglet there was a mild, multifocal, suppurative mesocolitis. In addition, in two of the piglets there was acute, mild, superficial, suppurative enteritis, with superficial necrosis and microthrombosis in one of the piglets. All four piglets also had mild atrophic enteritis.

### C. difficile toxin testing, isolation and PCR Ribotyping

Colonic contents were positive for *C. difficile* toxins A/B using using Premier Elisa Kit Toxins A&B (Meridian Bioscience Inc.)

Fifteen μL colonic contents were treated with 50 μL (96 %) ethanol. Fifteen μL of each mixture was transferred individually to plates containing Brazier’s cefoxitin cycloserine egg-yolk (CCEY) medium (Lister, 2014). Plates were incubated anaerobically (10%H_2_, 10%CO_2_ and 80%N_2_) at 34 °C for 48 h. ‘Broken glass’ colonies, typical of *C. difficile*, were transferred to blood agar plates and incubated for a further 48 h. Chelex-100 chelating resin was used for whole-cell DNA extraction of the resulting colonies. PCR-amplification of the DNA was performed with BioMix Red mastermix (Bioline) and CD-16s primers, 5′-CTG GGG TGA AGT CGT AAC AAG G-3′, 6′-GCG CCC TTT GTA GCT TGA CC-3′ (Eurofins MWG). PCR products were transferred to a heating block, set to 75 °C, for 45 min to concentrate products to c.20 μL, before electrophoresis on a 3 % agarose gel with GelGreen nucleic acid stain (Biotium). The gel was placed under ultraviolet light in a closed chamber of Bio-Rad Universal Hood II. Results were visualised with Bio-Rad Quantity One (4-6-1) 1-dimensional analysis software. PCR ribotypes were successfully obtained for three piglets; two were ribotype 078 and one was ribotype 110. The fourth piglet’s sample failed to yield a pure culture after treatment with 96 % ethanol and anaerobic incubation with CCEY medium.

### Other laboratory testing

*Clostridium perfringens* (*C. perfringens*) was isolated by direct anaerobic culture of intestinal contents using pre-reduced 5 % Columbia sheep blood agar (SBA) and fastidious anaerobe blood agar with nheomycin (E&O Laboratories, Scotland). *C. perfringens* alpha toxin was detected in a pooled sample of small intestinal contents using a sandwich ELISA, testing for alpha (α), beta (β), epsilon (ɛ) toxins and *C. perfringens Antigen* (BioX Diagnostics, Belgium).

Rotavirus group B was detected in small intestinal contents in two of the piglets using modified versions of previously described PCR methods [7, 8]. PCRs for porcine coronaviruses and porcine reproductive and respiratory virus were negative using modified versions of previously described methods [9–12]. No antigen was detected using anti-*Cryptosporidium parvum* monoclonal antibody labelled with fluorescein isothiocyanate (Bio-X Diagnostics, Belgium, Catalogue Number BIO 073).

## Conclusions

This is the first confirmed report of typhlocolitis associated with *C. difficile* in Irish pigs. *C. difficile* infection in the four piglets here appeared to be acting only as one component in a multifactorial diarrhoea that also involved Group B rotaviruses and possibly *Clostridium perfringens* Type A. Ongoing surveillance is required to determine the relative significance of *C. difficile* in porcine neonatal diarrhoea in Ireland.

This is also the first report of PCR-ribotyping of *C. difficile* isolates from clinically affected Irish pigs. There are a number of different molecular methods available for strain typing, most commonly polymerase chain reaction (PCR)-based ribotyping, multilocus variable number tandem repeat analysis (MLVA), pulsed field gel electrophoresis (PFGE) and whole genome sequencing (WGS) [13, 14]. PCR ribotype 078 is the most commonly reported isolate from pigs in most studies, including a recent Irish study in pigs of different ages [2, 14–16]. In one study in humans this strain was shown to have increased by at least 6-fold from 2000 to 2008 [17]. Moreover, genetically indistinguishable *C. difficile* 078 stains have been found in pigs and farmers, indicating interspecies transmission but the route of transmission has not been determined [18]. Therefore there is increasing interest in *C. difficile* as a ‘One Health’ issue. Our findings of two ribotypes in this one herd are consistent with previous reports of diversity of *C. difficile* ribotypes amongst pigs in an international study of animal associated strains, and a previous report from Germany [19, 20].

There are approximately 1600 new cases of human *C. difficile* infections per annum in Ireland, 21.5 % of which are recognised to be community-acquired infections i.e. patients had not had admission to a healthcare facility for 12 weeks preceding symptom onset [21]. A UK study has shown that only 38 % of CDAD in hospital in-patients can be attributed to transmissions within the hospital [22]. This has led to increased interest in the ‘One Health’ epidemiology of *C. difficile*. PCR ribotype results were available for only 16 % of human cases in Ireland in 2014: ribotypes 078 and 014 were most frequently reported, both 11 % of known results [21].

This report has confirmed that *C. difficile* is present in Irish pigs and is associated with typhlocolitis as part of multifactorial diarrhoea. Further studies are warranted to determine the prevalence of *C. difficile* in Irish pigs and other animal species. In addition, molecular typing studies using conventional methods such as PCR-based ribotyping or whole genome sequencing may provide information on any relationship that may exist between strains from animals and humans in Ireland.

## Abbreviations

CDAD: *Clostridium difficile*-associated disease
PCR: Polymerase chain reaction
